# Are Children Suffering From Congenital Pseudarthrosis of the Tibia Associated With Decreased Bone Strength?

**DOI:** 10.3389/fped.2022.859580

**Published:** 2022-05-09

**Authors:** Ge Yang, Siyu Xu, Haibo Mei, Guanghui Zhu, Yaoxi Liu, Qian Tan, Hui Yu

**Affiliations:** ^1^Department of Pediatric Orthopedics, The Hunan Children's Hospital, Changsha, China; ^2^Department of Orthopedics, Guangdong Provincial People's Hospital, Guangdong Academy of Medical Sciences, Guangzhou, China

**Keywords:** congenital pseudarthrosis of the tibia, bone quality, quantitative ultrasound, bone mineral density estimation, ultrasonic bone densitometer

## Abstract

**Background:**

Congenital pseudarthrosis of the tibia (CPT) is a rare and difficult-to-treat congenital disease in neonates. Our previous study found that exosomes derived from serum of children with CPT inhibit bone formation. In this study, we used ultrasound bone densitometry to detect the bone strength differences between hospitalized children with CPT and with non-metabolic diseases to determine the bone strength of children with CPT.

**Methods:**

A total of 37 children with CPT with a mean age of 3.14 ± 1.81 years and 40 hospitalized children with a mean age of 3.32 ± 2.66 years with supracondylar fracture of the humerus and without a bone metabolic disease (control group) were recruited in our hospital. The ultrasonic bone densitometer was used to examine the bilateral calcaneus of the subjects. We collected the broadband ultrasonic attenuation (BUA), speed of sound (SOS), quantitative ultrasound index (QUI), bone strength index (STI) and bone mineral density estimation (BMDe) values. Multivariable regression was used to examine the associations between quantitative ultrasound measurement differences and age, body mass index (BMI), neurofibromatosis type 1 (NF1) and CPT Crawford type. Intra-class correlation coefficient (ICC) was calculated to estimate intra- and inter-rater agreements.

**Results:**

74 calcaneus scans were taken from CPT patients (23 boys and 14 girls) and 80 calcaneus scans were taken from the control (24 boys and 16 girls). The CPT patients exhibited significantly lower SOS (1,368.75 ± 136.78 m/s), STI (7.2319 ± 38.6525), QUI (8.2532 ± 56.1720), and BMDe (−0.0241 ± 0.3552 g/cm^3^) than the control (SOS: 1,416.02 ± 66.15 m/s, STI: 7.96 ± 16.884, QUI: 28.8299 ± 25.461, BMDe: 0.0180 ± 0.1610 g/cm^3^). Multiple regression revealed that SOS, STI and QUI were statistically significant and negatively correlated with CPT Crawford classification.

**Conclusions:**

We found the incidence of decreased bone strength in CPT group was higher than that in the non-bone metabolic disease group. This phenomenon was not related to NF1 but related to CPT Crawford classification, which suggested that the higher the grade of the CPT Crawford classification, the lower the bone strength and the higher the risk of fracture.

## Introduction

Congenital pseudarthrosis of the tibia (CPT) is a rare and difficult-to-treat congenital disorder in newborns with an incidence of 1/140,000–1/250,000 (1, 2). It often presents with angular deformities, cysts, and epiphyseal stenosis. Patients with CPT are prone to re-fracture after spontaneous or minor trauma, heal poorly with conventional treatment, and eventually develop pseudarthrosis (3, 4). In order to maintain stability in pseudarthrosis, multiple operations are often required to achieve fracture healing (2, 3). Although surgical approaches and early healing rates have been improved, there are still problems such as high refracture rates, limb shortening and angulation, which pose serious challenges to clinical management (5–7).

Studies have shown that children with CPT are usually associated with neurofibromatosis type 1 (NF1), a disorder in which there is a loss of function of the NF1 gene, and CPT is also a rare manifestation of NF1 (8, 9). During skeletal development, the deletion of the NF1 gene decreases the level of bone metabolism, and children's bones exhibit excessive catabolic and anabolic deficiencies with a decreased level of 25-hydroxyvitamin D3 (25-OHD) (10). NF1 patients are also shorter than expected, indicating a general decrease in skeletal growth (11, 12). Research has shown that children and adults with NF1 have lower bone mineral density and exhibit less intense bone metabolism than normal (11–13). A previous study by our team found that exosomes derived from the serum of children with CPT associated with NF1 reduce bone formation (14). However, to date, there have been few clinical studies on bone metabolism levels in these patients, and it remains unclear whether bone metabolism in children with CPT differs from that of normal subjects.

Bone strength reflects the ability of a bone's resistance to fracture for a given condition, and bone stiffness index (STI) and bone mineral density estimation (BMDe) are important parameters for evaluating bone strength (15). Quantitative ultrasound is the most effective and widely used method for assessing bone strength in children (16). It is performed by measuring the tibia bone to obtain broadband ultrasound attenuation (BUA) and speed of sound (SOS) (16). The quantitative ultrasound index (QUI), STI and BMDe can be calculated by these two parameters (17). Compared with other BMDe testing methods, the quantitative ultrasound technique has the advantages of simplicity, convenience, no radiation, high accuracy, and low requirements for infant cooperation, so it is widely used for BMDe detection during the growth and development of children (16, 17).

As children with CPT are always associated with NF1 symptoms, and tibial pseudarthrosis is clinically difficult to cure, we hypothesized that these conditions may be caused by abnormalities in systemic skeletal metabolism. In this study, we used ultrasound bone densitometry to measure bone strength in children with CPT vs. hospitalized children suffering supracondylar fracture of the humerus without a metabolic bone disease to detect the bone strength in children with CPT.

## Materials and Methods

A cross-sectional study design was used to compare bone strength in CPT children with NF1 and hospitalized children without a bone metabolic disease. Informed consent was granted from all participants' parents. All data were analyzed after obtaining approval from the Ethics Committee of Hunan Children's Hospital.

### Participants

Thirty-seven children with a mean age of 3.14 ± 1.81 years with a diagnosis of CPT who had not received any previous treatment and 40 hospitalized children with a mean age of 3.32 ± 2.66 years suffering with supracondylar fracture of the humerus without a metabolic bone disease were included in this study. Participants were excluded if they had any known bone disease, other chronic diseases, previous or current treatment that might affect bone metabolism (e.g., celiac disease, thyroid disorder, systemic glucocorticoids), or if the child was unable to co-operate with the study protocol.

### Data Collection

All data were collected by the same researcher (S.X.), ensuring that all measurements were taken in the same way. General information was collected, including name, age, sex, and body mass index (BMI). The ultrasonic bone densitometer (Pegasus Smart Medilink, French) was used to examine the bilateral calcaneus of the subjects. The probe was placed correctly and faced the heel to avoid errors in the measurement results. The measured data were automatically analyzed by the computer system. According to the measurement method, we could obtain the BUA and SOS values, and then refer to the methods of calculating the QUI, STI and BMDe values (18, 19). To verify the reliability of the measurements, twenty subjects were randomly selected from the CPT and control groups and measured repeatedly by two of the authors (GY and SX) with an interval of 1 month to examine inter- and intra-rater agreements.

### Observation Index

Broadband ultrasound attenuation (BUA) (18, 19): Ultrasound waves are attenuated when passing through an elastic medium. During the measurement process, the transmitting probe of the bone densitometer emits ultrasound signals which are received and amplified by the receiving probe after passing through the bone sample to obtain the corresponding transmitted signals. The transmitted signals are processed accordingly to obtain the attenuation curve. The attenuation is linearly related to the frequency. The slope of the attenuation curve is BUA. The thicker the bone, the greater the bone density, the greater the attenuation of the signal at the same frequency, and the greater the corresponding slope. The BUA value is related to the average density, size, spacing, orientation and scattering intensity of the bone trabeculae. Due to low bone calcium density, patients with osteoporosis have lower BUA values than normal individuals.

Speed of sound (SOS) (18, 19): The ultrasound emission time and signal reception time were recorded and the SOS value was obtained based on the width of the sample. SOS is an indicator to evaluate bone condition and strength, and is proportional to bone volume.

Bone strength index (STI) (18, 19): According to the obtained results, STI = 0.67 × BUA + 0.28 × SOS – 420, which is an index obtained from the linear combination of BUA and SOS and mainly reflects the stiffness and rigidity of bone. Several studies have shown that it reflects both the mass and structural properties of cancellous bones. It has a better correlation and higher accuracy than bone mineral density. The index predicts the risk of osteoporotic fracture better than ultrasound velocity or attenuation parameters alone.

Quantitative ultrasound index (QUI) (18, 19): Statistical methods were based on previous descriptions (18), QUI = 0.41 × (BUA + SOS) −571.

Bone mineral density estimation (BMDe) formula (18, 19): Magkos' method (19) was used to convert BUA and SOS values into BMD values as follows: BMDe = 0.0025926 × (BUA + SOS) −3.687.

### Statistical Analysis

All data were expressed as mean ± standard deviation and processed by STATA (version 13.0, Stata Corp LP, TX, USA). The intraclass correlation coefficient (ICC) was calculated to estimate intra- and inter-rater agreement. According to Landis' definition, an ICC of 0.6–0.8 was considered good agreement and >0.8 was considered excellent agreement. Paired *t*-tests were used to determine within-group differences for various quantitative measures of quantitative ultrasound. For between-group analysis, *t*-tests were used to determine differences in various quantitative measures. Multivariate liner regression was used to investigate the relationship between differences in quantitative ultrasound measurements and age, BMI, NF1, and CPT Crawford type. *P* < 0.05 was considered statistically significant.

## Results

### Baseline Data and Measurement Reliability

In total, we reported 37 pairs of calcaneus scans from CPT patients and 40 pairs of scans from the controls. Among the CPT patients, 19 were Crawford type-II, and 18 were Crawford type-IV. The demographic characteristics of the CPT patients and controls are shown in Table 1. Reliability evaluation showed that all quantitative measurements had good to excellent intra- and interlocal agreement according to Landis' definition (Table 2).

**Table 1:** Baseline characteristics of CPT patients and controls.

	**CPT group (*n* = 37)**	**Control group (*n* = 40)**	***P***
Male	23	24	0.85
Female	14	16	
Mean age (year)	3.14 ± 1.81	3.32 ± 2.66	0.32
BMI (kg/m^2^)	15.47 ± 3.0	15.74 ± 3.75	0.53

**Table 2:** Reliability of quantitative ultrasound measurements.

**Measurement**	**Intra-rater agreement**		**Inter-rater agreement**	
	**ICC**	**95% CI**	**ICC**	**95% CI**
SOS	0.95	0.92, 0.98	0.97	0.94, 0.98
BUA	0.91	0.87, 0.93	0.92	0.90, 0.96
STI	0.96	0.92, 0.98	0.95	0.93, 0.96
QUI	0.97	0.93, 0.98	0.92	0.91, 0.94

*ICC, intra-class correlation coefficient; 95% CI, 95% confidence interval*.

### Quantitative Ultrasound Measurements

The quantitative ultrasound measurements between CPT patients and controls are presented in Table 3. Overall, the CPT patients exhibited significantly lower SOS, STI, QUI and BMDe than the controls (Table 3), whereas no significant differences were found in the BUA values between the CPT patients and controls (Table 3).

**Table 3:** Intragroup differences in the measurements of quantitative ultrasound.

**Measurement**	**CPT group (*****n*** **=** **37)**			**Control group (*****n*** **=** **40)**		
	**Affected**	**Contralateral**	***P***	**Left**	**Right**	***P***
SOS	1,371.67 ± 58.63	1,365.85 ± 185.70	0.85	1,412.41 ± 73.02	1,419.64 ± 59.2	0.52
BUA	43.01 ± 6.57	45.04 ± 8.84	0.18	46.1 ± 11.5	47.85 ± 13.08	0.31
STI	−7.08 ± 17.03	−7.39 ± 52.34	0.97	6.36 ± 18.6	9.56 ± 15.05	0.27
QUI	9.04 ± 24.22	7.46 ± 76.23	0.90	26.99 ± 28.29	30.67 ± 22.50	0.40
BMDe	−0.02 ± 0.15	−0.03 ± 0.48	0.90	0.09 ± 0.18	0.12 ± 0.14	0.40

For the above statistically significant parameters, we selected SOS, STI, QUI and BMDe as dependent variables and defined age, BMI, gender and NF-1 as independent variables. The final multiple linear regression model was not statistically significant and the regression coefficients of the measurements obtained by quantitative ultrasound with age, gender, BMI and NF-1 were not statistically significant (Table 4).

**Table 4:** Intergroup differences in the measurements of quantitative ultrasound.

**Measurement**	**CPT group (*n* = 74)**	**Control group (*n* = 80)**	***t***	***P***
SOS	1,368.75 (±136.78)	1,416.02 (±66.15)	2.7617	0.0065
BUA	44.0541 (±7.7963)	46.975 (±12.294)	1.7447	0.0831
STI	7.2319 (±38.6525)	7.96 (±16.884)	3.2013	0.0017
QUI	8.2532 (±56.1720)	28.8299 (±25.461)	2.9643	0.0035
BMDe	−0.0241 (±0.3552)	0.0180 (±0.1610)	2.9643	0.0035

We further analyzed the effects of age, sex, BMI, and Crawford classification of pseudarthrosis on SOS, STI, QUI, and BMDe. The final regression model showed that STI was positively correlated with age, while SOS, STI, and QUI were all negatively correlated with gender and CPT Crawford classification (Table 5).

**Table 5:** Associations of CPT differences parameters with age, gender, BMI and Crawford classification.

**Measurement**	**Age**		**Gender**		**BMI**		**II-Type**		**IV-Type**	
	**Coef**.	***P***	**Coef**.	***P***	**Coef**.	***P***	**Coef**.	***P***	**Coef**.	***P***
SOS	3.262	0.130	−32.517	0.009	6.63	0.065	−42.682	0.013	−91.495	0.000
STI	1.312	0.027	−9.84	0.004	1.522	0.119	−11.196	0.016	−28.473	0.000
QUI	1.581	0.068	−13.781	0.006	2.513	0.081	−17.037	0.013	−39.262	0.000
BMDe	−0.005	0.745	0.0051	0.956	0.033	0.224	0.011	0.931	−0.122	0.304

## Discussion

CPT is a rare disease in pediatric orthopedics. Once a fracture of the tibia occurs, it rarely heals naturally and usually results in a persistent pseudarthrosis. The treatment of CPT remains a surgical challenge, and fracture healing is difficult regardless of the surgical approach chosen (20). There are many factors behind a decreased ability of fracture healing and increased occurrence of refracture, but it remains unknown whether they are related to the bone strength of the whole body.

Recently, quantitative ultrasound has been introduced as a detection tool for orthopedic diseases, and its application in pediatric patients is also expanding (21). Previous research has shown that its detection rate of abnormal bone mass is significantly higher than that of the dual-energy X-ray detection method (18). Furthermore, the dual-energy X-ray detection method has the disadvantages of radiation exposure and difficulty in portability, which makes its use obviously limited.

In this study, quantitative ultrasound was used to detect the bone mineral density parameters of children with CPT and those with non-bone metabolic diseases. The incidence of osteopenia in the CPT group was higher than that in the control group, and the SOS, STI, QUI, and BMDe of CPT children were lower than those of children with non-bone metabolic diseases. SOS is an index for evaluating bone strength, which is proportional to bone mass and can better predict the risk of fracture (22). Therefore, our results suggest that the bone mass of children with CPT may be lower than those in the controls, and thus could be more prone to fracture. STI and QUI are skeletal strength indicators, which reflects the hardness and stiffness of bone (23). Our results indicate that the bone strength and bone stiffness of children with CPT may be lower than those of children with non-bone metabolic diseases. Although the BMDe values were not directly measured by ultrasonic bone densitometry, the BMDe values estimated by Magkos' method were statistically different between the two groups in our study, suggesting that the bone mineral density of children with CPT may also be lower than that of children without bone metabolic diseases. The above results indicate that the bone strength of children with CPT may be lower than that of similarly aged children without bone metabolic diseases, and there is bone loss, which may lead to increased bone fragility and easy fracture, and may also be associated with the high occurrence of refracture.

Studies have found that NF1 affects systemic bone metabolism (24, 25), and different clinical manifestations of pseudarthrosis are related to the occurrence of refracture (24, 26, 27). To investigate whether differences in bone strength are associated with clinical symptoms of pseudarthrosis and the presence or absence of NF-1, we performed multiple liner regression analyses of statistically significant quantitative ultrasound parameters with the presence or absence of NF-1 and CPT Crawford classification, respectively. Our study here shows that SOS, STI, QUI are negatively correlated with Crawford classification, suggesting that a higher level of CPT Crawford classification is associated with lower bone mass and strength, and thus could lead to a higher risk of bone fracture. However, there was no significant correlation between NF1 and SOS, STI, QUI, demonstrating that the presence or absence of NF1 has no significant effect on bone strength in children with CPT.

This study has some limitations. The above results obtained by cross-sectional studies require long-term follow-up, and the sample size may be small. In addition, children with CPT suffer from immobilization and pain of the affected limb which may lead to a reduction in their physical activity, and the change of bone mineral density may also be related to this. There is also a lack of activity scales to quantitatively assess children with CPT, thus, in future studies, we should recruit more subjects and identify more parameters to reduce the effects of the above impact factors. The design of an activity scale for children with CPT is also a topic for our future research.

## Conclusions

In conclusion, by recruiting children with CPT and those with non-bone metabolic diseases, we found that the incidence of osteopenia in the CPT group was higher than that in the non-bone metabolic disease group, and SOS, STI, QUI and BMDe in the CPT group were lower than in the non-bone metabolic disease group, and this phenomenon is not related to NF1 but is related to CPT Crawford classification, suggesting that a higher CPT Crawford classification is associated with lower bone strength and higher risk of fracture.

## Data Availability Statement

The original contributions presented in the study are included in the article/supplementary material, further inquiries can be directed to the corresponding author/s.

## Ethics Statement

The studies involving human participants were reviewed and approved by the Institutional Review Board of Ethics Committee of Hunan Children's Hospital (protocol code HCHLL-2021-129). Written informed consent to participate in this study was provided by the participants' legal guardian/next of kin. Written informed consent was obtained from the individual(s), and minor(s)' legal guardian/next of kin, for the publication of any potentially identifiable images or data included in this article.

## Author Contributions

GY and HM: conceptualization. HY: methodology. SX, GZ, and YL: validation. GY: formal analysis, investigation, data curation, and writing—original draft preparation. HM: resources, project administration, and funding acquisition. GY, HY, and HM: writing—review and editing. HM and HY: supervision. All authors have read and agreed to the published version of the manuscript.

## Funding

This research was funded by National Natural Science Foundation of China (Grant No. 82101818) and Key Research and Development Program of Hunan Province (2020SK2113).

## Conflict of Interest

The authors declare that the research was conducted in the absence of any commercial or financial relationships that could be construed as a potential conflict of interest.

## Publisher's Note

All claims expressed in this article are solely those of the authors and do not necessarily represent those of their affiliated organizations, or those of the publisher, the editors and the reviewers. Any product that may be evaluated in this article, or claim that may be made by its manufacturer, is not guaranteed or endorsed by the publisher.

## Abbreviations

CPT: congenital pseudarthrosis of the tibia
BUA: broadband ultrasound attenuation
SOS: speed of sound
STI: bone strength index
BMDe: bone mineral density estimation
NF1: neurofibromatosis type 1
ICC: intraclass correlation coefficient
BMI: body mass index
CI: confidence interval.
